# Supplementary material for: Content-rich biological network constructed by mining PubMed abstracts
Source: BMC Bioinformatics. 2004 Oct 8;5:147. doi: 10.1186/1471-2105-5-147 (PMC528731; doi:10.1186/1471-2105-5-147)
Supplement: Additional File 2 — The original results of the above study (non-essential files are deleted to keep the file size under the limit set by BMC bioinformatics). [file 1471-2105-5-147-S2.bz2 › chilibotAdditionalFile2/dip30/580ID8624812E10948/html/HLA_B2M.html]

 


 **HLA** and **B2M** 
  
Found 815 abstracts in PubMed, retrieved 30.  
 

 What does Google say? 
 PDF only 
| .edu only 

---

**Interactive relationship** (e.g. stimulation, inhibition, etc)

**Neutral relationship**- We cloned the extracellular domain of a human MHC class I heavy chain,  **HLA**  A\*2402, and human beta 2 microglobulin  [ **B2M** ]  beta2m fused with  **HLA**  A\*2402 restricted human immunodeficiency virus type 1 HIV 1 cytotoxic T lymphocyte CTL epitopes e beta2m in separate SeV vectors.  Ref: 12414940 J Virol, 2002
- We previously described Tg mice carrying the  **HLA**  B27,  **HLA**  B7, or  **HLA**  A2 alleles expressed as fully native  **HLA**  nat with human beta 2 microglobulin  [ **B2M** ]  and as hybrid human mouse  **HLA**  hyb molecules on the H2 b background.  Ref: 12421934 J Immunol, 2002
- Beta 2 microglobulin  [ **B2M** ]  protein expression was lost in five of six of the  **HLA**  I negative cases, but frame shift mutations in three repetitive sequences in beta2 microglobulin were absent.  Ref: 12480922 Lab Invest, 2002
- Class I  **HLA**  oligomerization at the surface of B cells is controlled by exogenous beta 2 microglobulin  [ **B2M** ]  implications in activation of cytotoxic T lymphocytes.  Ref: 12618477 Int Immunol, 2003

**Non-interactive relationship** (e.g. studied together, co-existance, homology, etc.)

- OBJECTIVE  **HLA**  B27 is capable of forming in vitro a heavy chain homodimer structure lacking beta 2 microglobulin  [ **B2M** ] .  Ref: 12428240 Arthritis Rheum, 2002
- We undertook this study to ascertain if patients with spondylarthritis express beta 2 microglobulin  [ **B2M** ]  free  **HLA**  B27 heavy chains in the form of homodimers and receptors for  **HLA**  B27 homodimers.  Ref: 12428240 Arthritis Rheum, 2002
- To explain the strong association between  **HLA**  B27 and ankylosing spondylitis, we suggest that the release of beta 2 microglobulin  [ **B2M** ]  beta 2 m from a subpopulation of cell surface expressed  **HLA**  B27 molecules leads to beta 2 m deposition within synovia and to the initiation of an inflammatory process, which culminates in destructive spondyloarthropathy.  Ref: 12547503 Trends Immunol, 2003
- The expression patterns of delta, tapasin,  **HLA**  Class I antigen, beta 2 microglobulin  [ **B2M** ] , and Survivin did not demonstrate any significant association with the clinical course of disease.  Ref: 12712472 Cancer, 2003
- The regions analyzed consisted of the  **HLA**  class I heavy chain genes located within the MHC genomic region on chromosome arm 6p, the  **HLA**  class I light chain beta 2 microglobulin  [ **B2M** ] ,  **B2M**  gene on chromosome arm 15q, and the putative  **HLA**  modifier of methylation gene MEMO1 located on chromosome arm 1q.  Ref: 12759931 Genes Chromosomes Cancer, 2003
- Expression of each  **HLA**  hyb molecule and  **HLA**  B27 nat human beta 2 microglobulin  [ **B2M** ]  led to peripheral CD8 T cell levels comparable with that for mice expressing a single H2 K b or H2 D b gene.  Ref: 12421934 J Immunol, 2002
- These regions comprise the 4Mb major histocompatibility complex MHC region on chromosome 6p, which contains the  **HLA**  class I heavy chain loci and other genes responsible for antigen processing, the  **HLA**  class I light chain beta 2 microglobulin  [ **B2M** ] , beta2m gene on chromosome 15q, and the putative  **HLA**  class I modifier of methylation gene MEMO 1 on chromosome 1p.  Ref: 12445306 Tissue Antigens, 2002
- Recombinant truncated forms of  **HLA**  B\*4405 1 276,  **HLA**  B\*4403 1 276,  **HLA**  B\*4402 1 276 and beta 2 microglobulin  [ **B2M** ]  have been prepared as inclusion bodies in Escherichia coli and refolded in the presence of the DPalpha 46 54 peptide and purified by a combination of size exclusion and anion exchange chromatography.  Ref: 12220628 FEBS Lett, 2002
- These cells were systematically characterized by Northern and or Western blotting e.g., mini transcriptome mini proteome analysis for the expression of  **HLA**  A, B, C, beta 2 microglobulin  [ **B2M** ] , and the members of the antigen processing machinery of class I molecules LMP2, LMP7, TAP1, TAP2, tapasin, calreticulin, calnexin, and ERp57.  Ref: 12874016 Cancer Res, 2003
- Rats on Lewis or Fischer background, transgenic for human  **HLA**  B27 and beta 2 microglobulin  [ **B2M** ]  genes spontaneously develop colitis, gastritis, arthritis, dermatitis, orchitis, epididymitis, carditis, alopecia and nail changes.  Ref: 12571416 Pathobiology, 2002
- The increase in cell surface expression of  **HLA**  I and beta 2 microglobulin  [ **B2M** ]  on infected fibroblasts was demonstrated by flow cytometric analysis.  Ref: 12379691 Infect Immun, 2002
- Here, the sensitivity of this homotypic association to exogenous beta 2 microglobulin  [ **B2M** ]  beta 2 m and the role of free heavy chains FHC in class I  **HLA**  oligomerization were investigated on a B lymphoblastoid cell line, JY.  Ref: 12618477 Int Immunol, 2003
- Beta 2 microglobulin  [ **B2M** ]  free  **HLA**  class I heavy chain FHC A3 and or A30 soluble products contribute only minimally to serum FHC expression.  Ref: 12358850 Eur J Immunogenet, 2002
- Loss of expression and mutations of the light chain of the  **HLA**  class I molecule, beta 2 microglobulin  [ **B2M** ]  beta 2 m have been described in a variety of human tumors and cell lines.  Ref: 12471623 Int J Cancer, 2003
- We previously showed that serum beta 2 microglobulin  [ **B2M** ]  beta2m free human leucocyte antigen  **HLA**  class I heavy chain FHC levels were increased in MM and correlate with disease activity.  Ref: 12492574 Br J Haematol, 2003
- In contrast to the high frequency of APM component down regulation,  **HLA**  class I heavy chain and beta 2 microglobulin  [ **B2M** ]  defects were detected in only 12 and 10% of the lesions, respectively.  Ref: 12738726 Clin Cancer Res, 2003
- Uniquely conformed peptide containing beta 2 microglobulin  [ **B2M** ]  free heavy chains of  **HLA**  B2705 on the cell surface.  Ref: 12370371 J Immunol, 2002
